# Evolution of Copper Homeostasis and Virulence in *Salmonella*

**DOI:** 10.3389/fmicb.2022.823176

**Published:** 2022-03-16

**Authors:** Andrea A. E. Méndez, Julián I. Mendoza, María Laura Echarren, Ignacio Terán, Susana K. Checa, Fernando C. Soncini

**Affiliations:** Instituto de Biología Molecular y Celular de Rosario, Facultad de Ciencias Bioquímicas y Farmacéuticas, Universidad Nacional de Rosario, Consejo Nacional de Investigaciones Científicas y Técnicas, Rosario, Argentina

**Keywords:** copper, bacterial envelope, CueP, CusCFBA, host-pathogen interaction

## Abstract

*Salmonella enterica* sv. Typhimurium modulates the expression of factors essential for virulence, contributing to its survival against the surge of copper (Cu) in the *Salmonella*-containing vacuole. This bactericidal host innate immune component primarily targets the bacterial envelope, where most cuproproteins are localized. While in most enteric species periplasmic Cu homeostasis is maintained by the CusR/CusS-controlled CusCFBA efflux system encoded in the *cus* locus, we noticed that these genes were lost from the *Salmonella*-core genome. At the same time, *Salmonella* acquired *cueP*, coding for a periplasmic Cu chaperone. As *cus*, *cueP* was shown to be essential for bacterial survival in a copper-rich environment under anaerobiosis, suggesting that it can functionally substitute the CusCFBA system. In the present study, the whole *Escherichia coli cus* locus was reintroduced to the chromosome of the *Salmonella* wild-type or the Δ*cueP* strain. While the integrated *cus* locus did not affect Cu resistance under aerobic conditions, it increases Cu tolerance under anaerobiosis, irrespective of the presence or absence of *cueP*. In contrast to the Cus system, CueP expression is higher at high copper concentrations and persisted over time, suggesting separate functions. Finally, we observed that, regardless of the presence or absence of *cus*, a mutant deleted of *cueP* shows a deficiency in replication inside macrophages compared to the wild-type strain. Our results demonstrate that CueP and CusCFBA exert redundant functions for metal resistance, but not for intracellular survival, and therefore for the virulence of this pathogen.

## Introduction

*Salmonella enterica* encompasses a zoonotic group of pathogens divided into seven subspecies and more than 2,600 serotypes (Alikhan et al., 2018). It is the causative agent of a variety of clinical ailments (from gastroenteritis to more serious systemic diseases) in both humans and animals, including those of economic relevance (Eng et al., 2015). The pathogen is acquired by ingestion of contaminated water or food and more rarely by direct contact with infected individuals (Branchu et al., 2018). Annually, almost 94 million cases of enteric salmonellosis and more than 150,000 deaths are reported worldwide. Most of the cases are self-limited and respond well to antimicrobial therapy. However, in young children, older adults, or immune-compromised patients, non-typhoid *Salmonella* can cause severe infections and sepsis (Eng et al., 2015; Branchu et al., 2018). This pathogen has a remarkable ability to adapt and survive to different harsh conditions, including the host environment. This is reflected by the versatility of its genetic repertoire (Alikhan et al., 2018; Branchu et al., 2018). Recent reports indicate that *Salmonella* detects the surge of copper (Cu) inside the *Salmonella*-containing vacuole (SCV) in infected macrophages, and mutants affected in terms of Cu resistance have a reduced intracellular survival compared to the wild-type strain (Achard et al., 2012; Osman et al., 2013; Fenlon and Slauch, 2017; Ladomersky et al., 2017).

Cu is not only an essential micronutrient but also a potent microbicidal agent (Borkow and Gabbay, 2005; Grass et al., 2011; Djoko and McEwan, 2013; Tan et al., 2017). Because of its ability to donate or accept one electron during Cu(I)/Cu(II) interconversion at a life-compatible redox potential, it was incorporated as a prosthetic group of many redox enzymes, being essential for aerobic growth, such as in cytochrome oxidases or superoxide dismutases (Rubino and Franz, 2012; Stewart et al., 2019). At the same time, the redox activity of the Cu(I)/Cu(II) pair contributes to its toxicity by catalyzing the generation of reactive oxygen species. Also, Cu ions bind with high affinity to S and N groups, affecting the structure and function of macromolecules as well as displacing other transition metals, such as Fe, from their binding sites, which exacerbates the redox stress (Macomber and Imlay, 2009; Djoko and McEwan, 2013; Le Brun, 2014; Tan et al., 2017). The toxicity of Cu has been exploited by eukaryotic cells to limit the growth of invading microorganisms, such as *Salmonella* (Besold et al., 2016). As part of their innate immunity, bacteria-infected macrophages increase the expression of membrane Cu transporters and their coupled chaperones to drive Cu trafficking and influx into the pathogen-containing phagosomes (Achard et al., 2012; Ladomersky et al., 2017). The ability to resist high Cu concentrations is crucial for virulence and involves factors localized to the cell envelope, the primary target of Cu toxicity. Various studies have shown that mutation of the two *Salmonella* Cu(I)-ATPases, CopA, and GolT, decreases survival inside RAW264.7 macrophages as well as in isolated peritoneal myeloid cells from C57BL/6J mice (Osman et al., 2010; Ladomersky et al., 2017). Interestingly, deletion of the gene coding for the *Salmonella*-specific periplasmic Cu chaperone, *cueP*, in *Salmonella enterica* sv. Typhimurium (*S*. Typhimurium hereafter) SL1344 strain also decreases its intracellular survival in macrophages (Yoon et al., 2014). The attenuated phenotype exhibited by these mutants depends on the functionality of the host Cu(I) ATPase ATP7A that delivers cytoplasmic Cu into the *Salmonella*-containing phagolysosomes (Ladomersky et al., 2017). Besides Cu-dependent redox imbalance, the ability of *Salmonella* to overcome the phagosomal oxidative burst also affects virulence (Negrea et al., 2009; Achard et al., 2010; Fenlon and Slauch, 2017; Yucel et al., 2020). This likely involves the redox activity of envelope cuproenzymes such as SodCI, SodCII, and CueO and the ScsABCD system of thioredoxins. Cells lacking SodCI or SodCII are less virulent (Fang et al., 1999), and virulence attenuation was also noticed for the Δ*cueO* or the Δ*scsC* strains (Achard et al., 2010; Yucel et al., 2020).

Most known bacterial cuproproteins localize to the cell envelope, making this compartment the main target of Cu toxicity (Rubino and Franz, 2012; Pontel et al., 2015; Giachino and Waldron, 2020; Checa et al., 2021). While most enteric species rely on CueO, the multicopper oxidase controlled by the cytoplasmic sensor/regulator CueR, to maintain periplasmic Cu homeostasis under aerobic conditions and on the CusR/CusS-controlled CusCFBA efflux system under anaerobic conditions, the *cus* locus is absent in the *Salmonella* genome (Checa et al., 2021). An *in silico* analysis revealed the presence of variable remnants of the outmost *cus* genes in most *Salmonella* strains, suggesting different deletion events during this species evolution (Checa et al., 2021). At the same time or probably before *cus* deletion, *Salmonella* acquired *cueP* (Pontel and Soncini, 2009). Interestingly, *cueP* transcription depends on the coordinated action of CueR, the ancestral Cu-responsive CueR regulator that also controls the expression of *copA* and *cueO* and of CpxR/CpxA, a main two-component system responding to multiple envelope stresses, including Cu and redox oxidative species (Pezza et al., 2016). Thus, CueP-induced expression occurs only under conditions of Cu stress that affect envelope homeostasis. Previously, we showed that, expressed from a multicopy plasmid, CueP can partially complement a Δ*cus Escherichia coli* strain for Cu resistance under anaerobic conditions (Pontel and Soncini, 2009), although these Cu resistance determinants are not structurally or functionally related. CusCFBA is a Cu^+^-specific envelope detoxification pump (Franke et al., 2003), while CueP is the major periplasmic cuproprotein, with a putative Cu^2+^ reductase activity (Osman et al., 2010, 2013; Yoon et al., 2013, 2014; Abriata et al., 2014). The phenotype analyses of a *S.* Typhimurium Δ*cueP* strain also mimics the *E. coli cus* deletion mutant in (i) its requirement for Cu resistance under anaerobic condition, (ii) the absence of an appreciable phenotype in aerobiosis, (iii) their delayed expression compared to the canonical CueR-regulated *copA* gene, and (iv) their coordinated transcriptional control to specifically respond to a cell-envelope-toxic Cu surge (Outten et al., 2001; Pontel and Soncini, 2009; Pontel et al., 2010; Fung et al., 2013; Pezza et al., 2016). Considering these observations and the proposed functional redundancy between CueP and the CusCFBA system, here we tested the hypothesis that the *cus* locus was selectively lost from *Salmonella* because either it is not required for intracellular survival or it interferes with virulence.

In this study, we reintroduced the *E. coli cus* locus in the identified *cus* scar present in the *S.* Typhimurium genome and evaluated its transcriptional profile and its role in Cu resistance and in virulence, both in the presence and absence of *cueP*. Although the Cus system is expressed in response to Cu in *Salmonella* and conferred high levels of Cu tolerance particularly under anaerobic conditions, we found that, in contrast to CueP, it did not contribute to intracellular survival in macrophages. These results indicate that, although CueP and CusCFBA exert redundant functions for Cu resistance, they are not exchangeable for macrophage survival and therefore for *Salmonella* virulence.

## Materials and Methods

### Bacterial Strains and Growth Conditions

The *E. coli* and *S.* Typhimurium strains and plasmids are listed in Supplementary Table 1. The cells were grown overnight at 37°C in Luria–Bertani broth (LB) with shaking or in LB agar plates. Kanamycin (Km) was used at 25 μg ml^–1^, chloramphenicol (Cm) at 10 μg ml^–1^, spectinomycin (Sp) at 50 μg ml^–1^, and ampicillin (Amp) at 100 μg ml^–1^. Bacterial stocks were stored at −80°C with 15% glycerol. A final concentration of 0.1 mM isopropyl β-D-1-thiogalactopyranoside was added when indicated to express CueP from a plasmid. The culture media was from Difco, whereas the rest of the reagents and chemicals were from Merck and affiliates. The copper salt used was of ACS analytical grade at ≥98.0% purity. The oligonucleotides were provided by Life Technology and are listed in Supplementary Table 2.

### Genetic and Molecular Biology Techniques

Insertion of the *cus* locus into the *S.* Typhimurium chromosome was carried out after two sequential steps of Red-mediated recombination protocol (Karlinsey, 2007). Briefly, a ∼4,900-bp fragment containing a Cm^R^–*cusRS*–*cusCF* region (product I) was amplified from the chromosome of the recombinant *E. coli* strain PB1179 (Supplementary Table 1) using the Q5^®^ High-Fidelity DNA polymerase (New England Biolabs) and the oligonucleotides Cus SF P1 Fw and Cus SF P2 Rv (Supplementary Table 2). The purified final product was introduced by electroporation into *S*. Typhimurium 14028s carrying the pKD46 plasmid (Supplementary Table 1). After selection of chloramphenicol-resistant colonies, proper insertion of product I was verified by colony PCR using the oligonucleotides detailed in Supplementary Table 2 in order to select strain PB13957 (Supplementary Table 1). In parallel, a second ∼6,100-bp fragment containing the final portion of cusF and the *cusBA*:3xFLAG-Km^R^ region (product II) was PCR-amplified from *E. coli* PB1179 (Supplementary Table 1) using the oligonucleotides P1 Fwd CusA Flag Km and Cus FA P2 Rv (Supplementary Table 2). After purification, product II was used to transform the PB13957 strain carrying pKD46. Kanamycin- and chloramphenicol-resistant colonies were selected to verify proper product II insertion following product I through colony PCR using the oligonucleotides detailed in Supplementary Table 2. After selecting one clone, the presence of the whole *E. coli cusRS*–*cusCFBA* locus into the *Salmonella* chromosome was verified by DNA sequencing at Macrogen Inc. P22-mediated transduction (Checa et al., 2007) was used to move the whole *cus* locus into the chromosome of the wild-type 14028s to obtain the PB14006 strain or to the chromosome of strains carrying the *cueP*:3xFLAG gene or the Δ*cueP*, Δ*cueO*, Δ*cueO*Δ*cueP*, or Δ*golT* Δ*copA* mutant strains (Supplementary Table 1).

Reporter plasmids pP*cueP*-*gfp* and pP*cusCFBA*-*gfp* (Supplementary Table 1) were constructed as follows: The *cueP* or the *cusABFC* promoter region was amplified by PCR from the chromosome of the PB14006 strain using the oligonucleotides listed in Supplementary Table 2. The product containing the *cueP* promoter was *Sma*I-digested and cloned into pPROBE-OT′ (Supplementary Table 1) digested with this enzyme. Similarly, the *cusCFBA* Inc., Hercules, CA, United States promoter was digested with *Hin*dIII/*Eco*RI enzymes and cloned into *Hin*dIII/*Eco*RI-digested pPROBE-OT′.

### Fluorescence Determination

A 100-μl aliquot of 1/100 overnight culture of the indicated strains grown in 96-well microplates in LB supplemented without or with 1, 2, 3, or 4 mM CuSO_4_ was incubated overnight at 37°C with regular shaking. Fluorescence (485-nm excitation/508-nm emission) and optical density (OD_600*nm*_) were determined from pP*cueP*-*gfp* or pP*cusCFBA*-*gfp* harboring *Salmonella* strains using a BioTek™ Sinergy™ HT Microplate reader every 1 h for a period of 16 h and used to calculate the normalized fluorescence expressed as arbitrary units. To prevent dehydration, the perimeter wells were filled with sterile water. Wells containing only culture media with/without CuSO_4_ were included as controls of background fluorescence.

### Western Blot Analysis

Western blot analysis of 3xFLAG-tagged proteins, IgaA or GroEL, were carried out as described previously (Pontel and Soncini, 2009; Pérez Audero et al., 2010). Briefly, cells were grown in the presence of 2 mM CuSO_4_ until OD_600*nm*_ of 0.5, harvested by centrifugation at 3,500 *g* for 10 min, washed, and resuspended in 1 ml of Tris-EDTA buffer solution (pH 8) supplemented with 1 mM phenylmethylsulfonyl fluoride. The cell suspensions were sonicated (30% amplitude) on ice for 2 min, with on/off intervals of 2 s. The mixtures were then centrifuged at 12,000 *g* for 30 min at 4°C to separate soluble and insoluble (membrane) fractions and determine the protein concentration. Aliquots of the soluble or insoluble fraction containing 20 or 10 μg of total proteins were analyzed in 15% (w/v) and 10% (w/v) sodium dodecyl sulfate polyacrylamide gels, respectively, and transferred to nitrocellulose membranes. Both the soluble CueP-3xFLAG and the membrane-bound CusA-3xFLAG proteins were detected using mouse anti-FLAG monoclonal antibodies (Sigma-Aldrich) and mouse secondary antibody conjugated with horseradish peroxidase (HRP). In parallel, rabbit polyclonal anti-GroEL or anti-IgaA antibodies and the specific secondary antibody conjugated with HRP were employed to detect the loading controls in the soluble and insoluble cell fractions, respectively. Immunoreactive bands were revealed using SuperSignal^®^ West Femto Maximum Sensitivity Western Blotting Substrate (Thermo Fisher Scientific Inc., Waltham, MA, United States) and registered in ChemiDoc™ XRS Imaging System (Bio-Rad Laboratories Inc., Hercules, CA, United States). A densitometric analysis of each band was done using the Gel-Pro software and used to estimate the amount of CueP-3xFLAG or CusA3xFLAG in the samples after normalization against the soluble and insoluble loading controls, GroEL or IgaA, respectively.

### Copper Resistance Assays

Minimum inhibitory concentrations (MICs) were determined in LB agar plates supplemented with CuSO_4_ at the indicated concentrations as previously described (Pontel and Soncini, 2009). Plates were incubated for 24 h at 37°C under aerobic condition or for 72 h at 37°C under anaerobiosis inside a jar containing Oxoid™ AnaeroGen™ System and Oxoid™ Anaerobic Indicator (Thermo Scientific). After incubation, the plates were photographically recorded, and the MIC values were registered.

### Intramacrophage Proliferation Assays

*Salmonella* proliferation in RAW 264.7 macrophages was tested as described (Echarren et al., 2021). Briefly, macrophages were cultured in 24-well plates containing Dulbecco’s modified Eagle’s medium (DMEM) supplemented with 10% fetal calf serum. Each *S.* Typhimurium strain tested was grown overnight, harvested, and washed with 1× phosphate-buffered saline (PBS). These bacterial pellets were resuspended in DMEM media and used for cell infection assay at a multiplicity of infection of 10 bacteria per cell at 37°C for 30 min. Afterward, fresh DMEM and 10% FBS medium supplemented with gentamicin (100 μg/ml) was added. After 1 h at 37°C, the infected cells were incubated with a medium containing gentamicin at a concentration of 30 μg/ml for a total of 18 h. At the indicated time points, the cells were washed and lysed with 0.1% Triton X-100 in PBS. Lysates were recovered, serially diluted, and spread on LB agar plates. After overnight incubation at 37°C, colony-forming units were counted and used to calculate the intracellular proliferation relative to the wild-type strain.

## Results

### The *Escherichia coli cus* Locus Is Transcriptionally Induced by Copper in *Salmonella* Typhimurium

**FIGURE 1:**
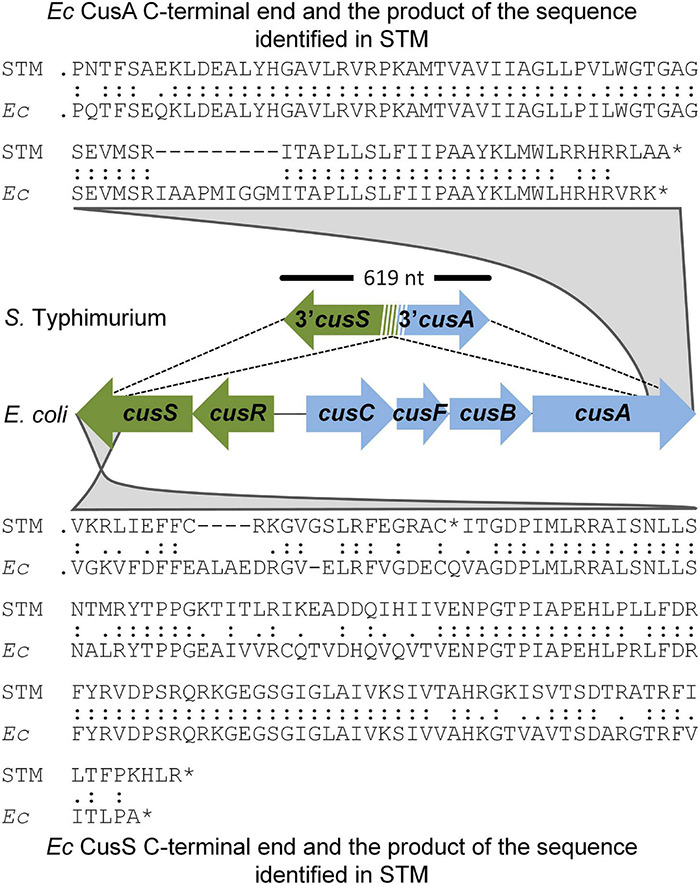
*Cus* remnants in the *Salmonella* Typhimurium chromosome. The figure shows the 619-bp region harboring remnants of the outermost ends of the *cus* locus identified in the *S*. Typhimurium (STM) genome and the homology with the *Escherichia coli (Ec)* CusA and CusS C-termini.

Early in evolution, *S. enterica* acquired *cueP*. This probably accompanied or presided by different *cus* locus deletion events, resulting in variable remnants of the outmost *cus* genes among different *S. enterica* serovars (Checa et al., 2021). As an example, the *S.* Typhimurium 14028s genome harbors a 619-bp DNA fragment, including sequences coding for the last 137 amino acids of CusS (with 65% identity) and the last 83 residues of CusA (with 83% identity) between nucleotides 619898 and 619287 (Figure 1), that is, only the C-terminal portion of both gene products, encoded in opposite directions in the ancient *E. coli cus* locus, remains in the *S*. Typhimurium genome. Interestingly, the residual fragments of *cusS* and *cusA* overlap (Figure 1), suggesting a site-specific recombination event.

**FIGURE 2:**
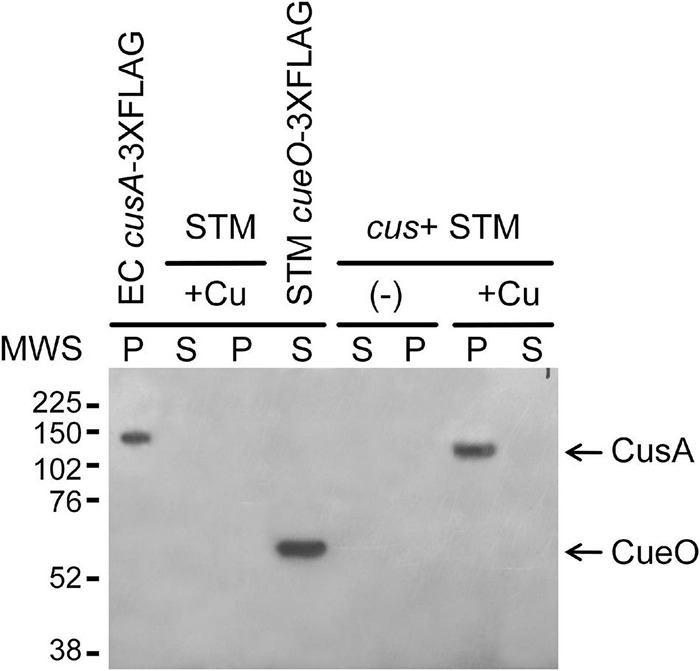
The *cus*+ *Salmonella* Typhimurium strain expresses CusA-3xFLAG in the presence of copper. Western blot analysis of soluble (S) and pellet cell fractions (P) of the transgenic *cus*+ *S*. Typhimurium strain grown overnight in the presence (+Cu) and absence (−) of 2 mM CuSO_4_. The pellet fraction of the W3110 *cusS*-Cm^R^ *cusA*:3xFLAG-Km^R^ *Escherichia coli* strain and the soluble fraction of the *cueO*:3xFLAG *S*. Typhimurium strain were used as positive controls, while the parental *S*. Typhimurium 14028s soluble and insoluble cell fractions were included as negative control. Anti-mouse FLAG monoclonal antibodies were used for the 3xFLAG immunodetection.

To analyze whether the *E. coli* CusCBA efflux pump and its associated CusF Cu chaperone can substitute CueP for Cu resistance and virulence in the *Salmonella* envelope, the *E. coli cus* locus was inserted into the *S*. Typhimurium 14028s *cus* scar (Supplementary Figure 1). We included a 3xFLAG-tag coding sequence at the *cusA* 3′ end to determine its CusR/CusS-dependent expression in response to Cu ions and, in parallel, to verify if the fusion protein is directed into the *S.* Typhimurium inner membrane. As expected, CusA-3xFLAG was detected in the insoluble cell fraction of the *cus*+ strain and only after CuSO_4_ addition to the culture medium, as occurs in *E. coli*, included as a control (Figure 2). No immunoreactive bands were detected in cell extracts from the wild-type *S*. Typhimurium strain.

### Copper-Dependent Transcriptional Induction of *cueP* and the *cusCFBA* Operon Occurs at Different Stages of Growth

**FIGURE 3:**
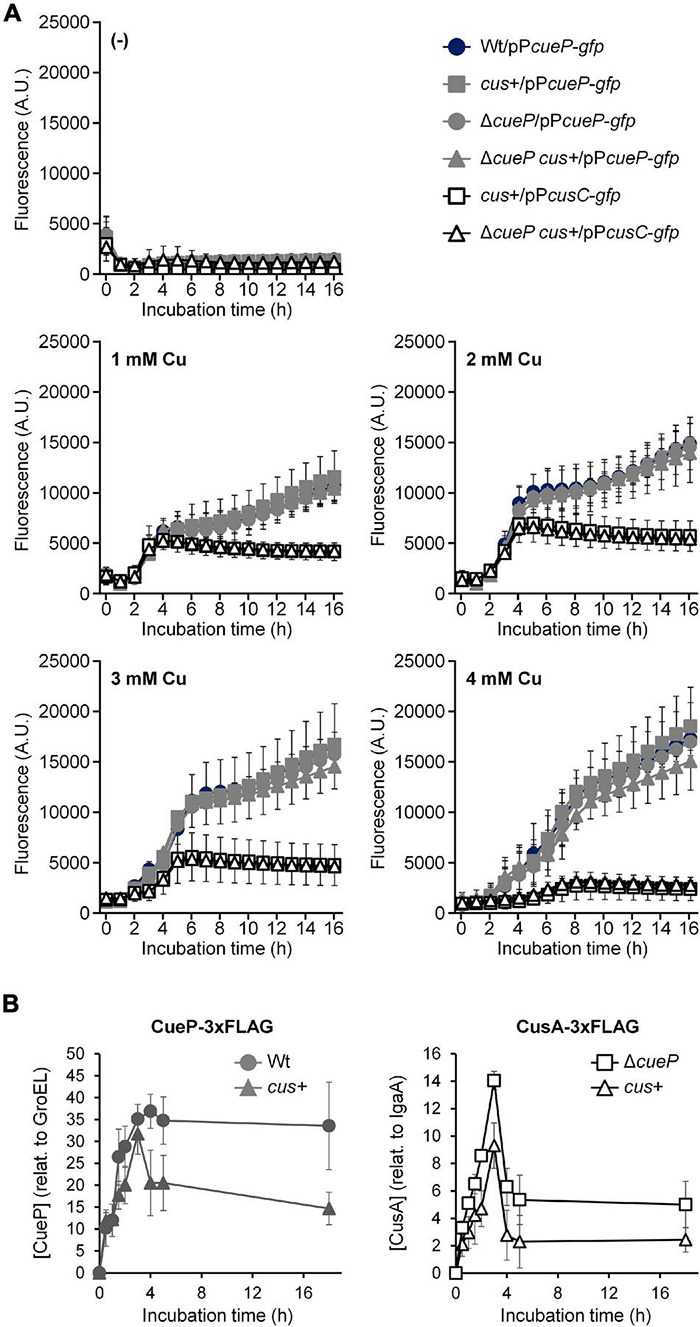
Comparative analysis of the Cu-induced expression of the *cus* locus and *cueP*. **(A)** Kinetic analysis of the Cu-induced fluorescence from the wild type, the *cus*+, the Δ*cueP*, or the Δ*cueP cus*+, harboring *gfp* transcriptional fusions to either P*cueP* or the P*cusC* promoter, as indicated. Either 0, 1, 2, 3, or 4 mM CuSO_4_ was added to cells exponentially grown in Luria–Bertani broth (LB), and both fluorescence and OD_600*nm*_ (see Supplementary Figure 3) were recorded for 16 h and used to calculate the normalized fluorescence expressed as arbitrary units. The data correspond to mean values of at least three independent experiments performed in duplicate. Error bars represent SD. **(B)** Kinetic analysis of the Cu-induced CueP-3xFLAG or CusA-3xFLAG expression from the *cueP*-3xFLAG (Wt), the *cus*+ *cueP*-3xFLAG *cusA*-3xFLAG (*cus*+), or the *cus*+ *cusA*-3xFLAG Δ*cueP* (Δ*cueP*) strains. Soluble or insoluble extracts from cells grown in LB with the addition of 2 mM CuSO_4_ were analyzed by SDS/PAGE, followed by transfer to nitrocellulose, and developed using monoclonal anti-FLAG antibodies as described in the Materials and Methods Section. CueP or CusA relative levels were normalized to GroEL or IgaA, respectively. The data correspond to mean values of three independent experiments. Error bars represent SD. A representative western blot is shown in Supplementary Figure 4.

To compare *cueP* and *cusCFBA* transcription in *S.* Typhimurium, the wild-type, its Δ*cueP* derivative, or the transgenic *cus*+ or *cus*+ Δ*cueP* strains were transformed with pP*cueP*-*gfp* or pP*cusC*-*gfp*, and fluorescence was recorded every hour during 16 h after the addition of different concentrations of Cu to the culture. After a lag period of ∼1 h, fluorescence increased in cultures from the wild-type strain harboring the pP*cueP*-*gfp* reporter plasmid supplemented with CuSO_4_ (Figure 3A). At low or intermediate Cu concentrations (1–2 mM CuSO_4_), P*cueP*-dependent GFP expression increased for about 4 h, reaching a plateau that persisted for another hour. After that, a new increase in fluorescence was observed, which continued at least during the 16 h that the experiment was recorded (Figure 3A). The plateau was less evident at 3 mM CuSO_4_ and disappeared at 4 mM CuSO_4_, the higher concentration tested. This expression profile could reflect the need for CueP in conditions of persistent Cu stress and/or at the stationary phase when other toxic species are expected to accumulate. At 1 or 2 mM CuSO_4_, no significant differences in emitted fluorescence were perceived between cells harboring *cueP* or the transgenic *cus* locus or not, although a lower P*cueP*-*gfp* promoter expression was evident from *cus*+ cells exposed to 3–4 mM CuSO_4_ (Figure 3A).

In contrast to the abovementioned observations, the CusR/CusS-dependent GFP expression from the *cusC* promoter was evident in the *cus*+ strain during exponential growth and particularly at low or intermediate CuSO_4_ concentrations, but it decreased at the stationary phase (Figure 3A). Interestingly, less induction from P*cusC* was observed at higher Cu concentrations, while this was the condition for maximal fluorescence from the P*cueP*-*gfp*-expressing strain. As with P*cueP*-*gfp*, we did observe any significant differences in P*cusC-gfp* expression between the strains bearing *cueP* and those not (Figure 3A). As expected, no fluorescence was detected from the wild-type strain or its Δ*cueP* derivative carrying the pP*cusC-gfp* reporter but lacking the whole *cus* locus in their chromosomes, indicating that Cu-dependent induction of the *cusCFBA* promoter requires CusR/CusS (Supplementary Figure 2). On the other hand and irrespective of the presence or absence of a functional *cueP* and/or *cus*, no differences in growth were detected in these strains even at 4 mM CuSO_4_ (Supplementary Figure 3), suggesting that the stress caused by the metal ion is managed by the innate aerobic Cu resistance apparatus primarily composed of the CueR- and GolS-dependent CopA, GolT, and CueO factors (Espariz et al., 2007; Pontel and Soncini, 2009; Pontel et al., 2010, 2014).

In view of these results, we decided to analyze the accumulation of CueP-3xFLAG and CusA-3xFLAG in the *cus*+ strain at different time points after Cu exposure to evaluate the effect of the simultaneous presence of both Cu resistance determinants on their reciprocal expression (Figure 3B and Supplementary Figure 4). Both the wild-type and the *cus*+ Δ*cueP* strain were included as controls. An increased accumulation of CueP-3xFLAG was detected in the otherwise wild-type strain for the first 3–4 h in cells exposed to 2 mM CuSO_4_ (Figure 3B). The maximal concentration reached during that period persisted at least up to 18 h. Interestingly, the tagged protein accumulated to similar levels in cells harboring the *cus* locus during the first 3 h, but a significant reduction was observed at longer times (Figure 3B). By contrast, CusA-3xFLAG increased its concentration in the membrane fraction of the recombinant *cus*+ Δ*cueP* strain and reached a maximal level at 3 h. This was followed by a decrease of the immunoreactive band, probably due to degradation of the membrane protein (Figure 3B and Supplementary Figure 4). Similar to CueP-3xFLAG, a consistent reduction of CusA-3xFLAG was visualized in cells also expressing CueP-3xFLAG. This clearly indicates that, at least at the protein level, the simultaneous presence of both components favors a reduction in the quantity of each individual system. In other words, these results are consistent with a functional redundance between the Cus system and the innate *Salmonella* CueP chaperone.

### The CusCFBA System Confers Higher Copper Resistance Levels Than CueP in Anaerobiosis

As reported in *E. coli* (Outten et al., 2001), the presence of the *cus* locus in *Salmonella* did not affect Cu resistance under aerobic conditions, even in cells lacking *cueP* (Supplementary Figure 5 and Supplementary Table 3). Because CueO is the main Cu(I) cell envelope detoxification factor when O_2_ is available (Espariz et al., 2007), the effect of *cusRS-CFBA* acquisition in *Salmonella* was tested in the Δ*cueO* background, both in the presence and absence of *cueP*. As previously reported for this genetic background (Pontel and Soncini, 2009; Pontel et al., 2010), CueP had only a minimal contribution to Cu tolerance under these conditions (Supplementary Figure 5). Surprisingly, the Δ*cueO cus*+ transgenic strain showed an increased resistance to the metal compared to the Δ*cueO* strain (Supplementary Table 3). A similar resistance phenotype with the Δ*cueO cus*+ strain was observed for the Δ*cueP*Δ*cueO cus*+ strain (Supplementary Figure 5), indicating that CueP has no impact on Cu resistance under these conditions. On the other hand, the presence of *cus* did not increase the Cu tolerance of a *Salmonella* strain deleted of both inner membrane-associated Cu(I) transporters *copA* and *golT*-coding genes (Supplementary Figure 5 and Supplementary Table 3), indicating that CusCFBA cannot alleviate the cytoplasmic toxic effect of Cu (Espariz et al., 2007). These results suggest that, despite its low expression under aerobiosis (Figure 3), CusCFBA can alleviate the toxic effects of Cu from the cell envelope in cells lacking the main Cu resistance determinant, CueO.

**FIGURE 4:**
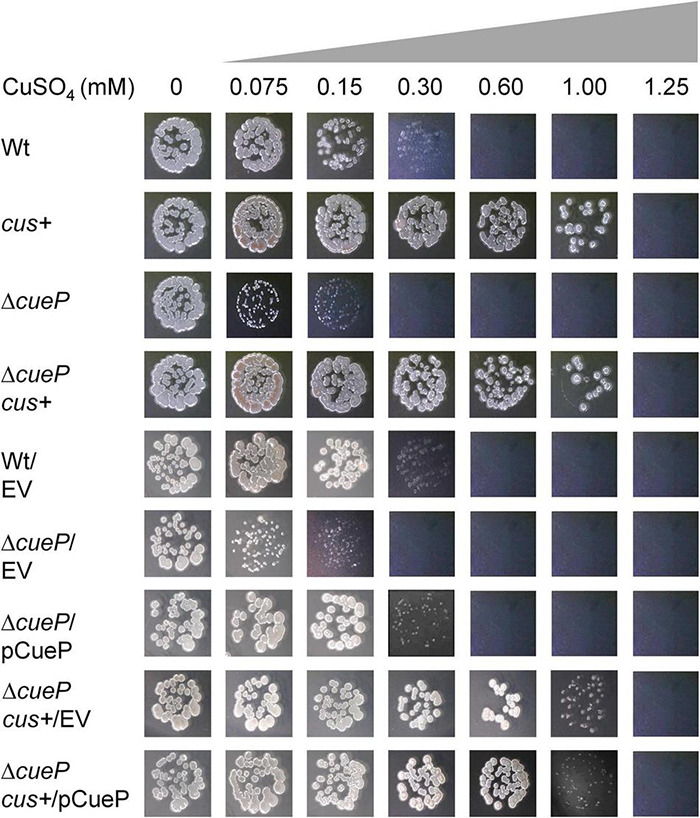
The *Escherichia coli cus* locus increases *Salmonella* tolerance to Cu under anaerobic conditions. Comparative Cu MIC values of wild type (Wt), transgenic *cus*+, Δ*cueP*, or Δ*cueP cus*+ *Salmonella* strains on Luria–Bertani broth plates containing increasing amounts of CuSO_4_ in anaerobic conditions. The figure also shows the same strains harboring the vector plasmid pUH21-2*lacI^q^* (EV) or pUH21-2 *lacI^q^*-based pCueP plasmid (pCueP). After incubation at 37°C for 24 h, the plates were photographed. The data correspond to representative images of at least three independent experiments done in duplicate.

As both CueP and CusCFBA were reported to contribute to Cu resistance under anaerobic conditions (Outten et al., 2001; Pontel and Soncini, 2009), we compared the tolerance to Cu of the transgenic *S*. Typhimurium *cus*+ strain, both in the presence and absence of *cueP* (Figure 4 and Supplementary Table 3). The presence of a functional CusCFBA system increased the Cu tolerance in these conditions up to 1 mM CuSO_4_, even in the Δ*cueP* strain. These strains were at least three times or six times more resistant than the wild-type strain or the Δ*cueP* mutant (Figure 4).

Not only is the CusCFBA system more efficient than CueP to eliminate toxic Cu ions, but also, in its presence, CueP turns non-essential even when it is overexpressed in the cells (Figure 4). Thus, why has *Salmonella* lost the beneficial *cus* locus while preserving *cueP*? We can speculate that niches normally encountered by this species would not simultaneously contain such a high Cu concentration and the absence of O_2_. Otherwise, the pathogen would retain the ancestral Cu–envelope homeostasis system. The acquired *cueP* gene product could fulfill the necessary metal resistance encountered by *Salmonella* in those particular niches. In this sense, the required co-regulation of *cueP* transcription, recruiting simultaneously the cytoplasmic Cu sensor CueR and the non-specific CpxR/CpxA envelope stress system, integrates different envelope stress signals, such as Cu and redox stress (Pezza et al., 2016; Cerminati et al., 2017; Grabowicz and Silhavy, 2017; Lopez et al., 2018; Subramaniam et al., 2019), that could also be beneficial in these niches.

### The Ancestral *Cus* System Does Not Contribute to *Salmonella* Intracellular Macrophage Proliferation

**FIGURE 5:**
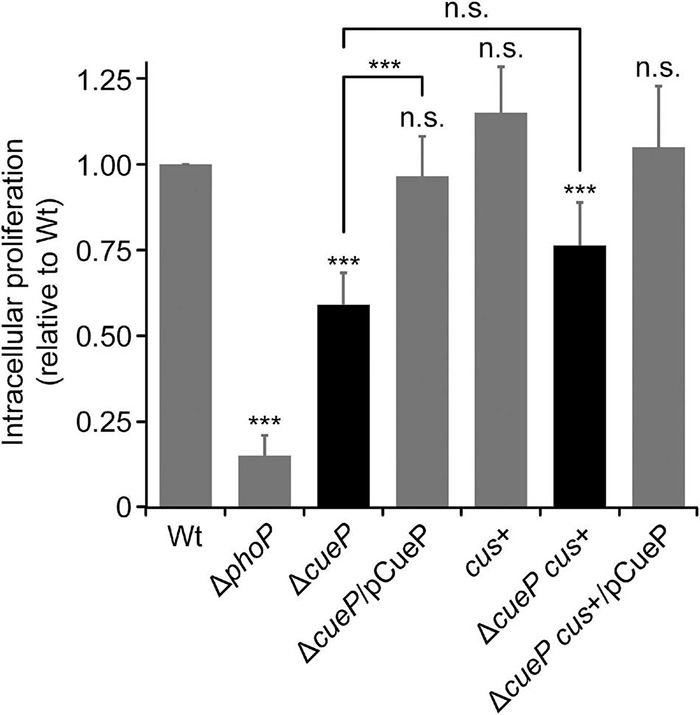
*Cus* cannot substitute *cueP* for intramacrophage proliferation. Replication of wild type (WT), Δ*cueP*, *cus*+, or Δ*cueP cus*+ *Salmonella* Typhimurium strains in RAW 264.7 macrophages at 18 h after infection. The virulence-defective Δ*phoP* strain was included as control. Complementation of Δ*cueP* or Δ*cueP cus*+ strains with pCueP is also shown. The values correspond to the average of at least three independent experiments carried out in duplicate, and the error bars represent SD. The asterisks denote statistical significance between means or with respect to WT. n.s., not significant; ***P < 0.001.

The SCV is known to be enriched in Cu ions and other toxic compounds, such as reactive oxygen/nitrogen species, that are actively delivered or produced by the host cell to eliminate invading pathogen (Negrea et al., 2009; Achard et al., 2010, 2012; Fenlon and Slauch, 2017; Ladomersky et al., 2017; Yucel et al., 2020). Knowing that a mutant deleted in *cueP* has a defect in macrophage proliferation (Yoon et al., 2014) and in view of the increased tolerance to Cu of the transgenic *S*. Typhimurium *cus*+ strain, we compared the intracellular proliferation of the latter strain with its Δ*cueP* derivative inside RAW 264.7 macrophages (Figure 5). As expected, the Δ*cueP* strain exhibited an attenuated phenotype compared to the wild type inside these professional phagocytes, while wild-type proliferation was reestablished by providing *cueP in trans*. The *S*. Typhimurium *cus*+ strain show wild-type levels of macrophage proliferation, indicating that its presence does not provide any advantage for survival in this environment (Figure 5) (It is worth noting that this strain harbors its wild-type chromosomal copy of *cueP*).

Surprisingly, the *cus*+ Δ*cueP* strain was as defective as the Δ*cueP* strain to proliferate inside this cell line, indicating that the Cus system cannot substitute CueP for *Salmonella* proliferation inside macrophages (Figure 5).

These results altogether indicate that the acquisition of *cueP* by *Salmonella* provides this pathogen with the ability to better replicate inside macrophages, and at the same time, it allows this species to tolerate moderate levels of Cu when facing oxygen limitation and other toxic species, whereas the Cus system cannot. These results indicate that CueP and CusCFBA exert redundant functions for metal resistance, but not for macrophage survival, and therefore for *Salmonella* virulence.

## Discussion

It is increasingly evident that, in Gram-negative species, the cell envelope is the primary target for Cu toxicity (Giachino and Waldron, 2020; Checa et al., 2021). It is in this compartment where all known Cu-requiring enzymes, such as multi-copper oxidases, amine oxidases, Cu-dependent superoxide dismutases, and terminal respiratory oxidases, are localized and where Cu-dependent metabolism occurs (Rubino and Franz, 2012; Stewart et al., 2019). Most enteric species rely on the periplasmic multicopper oxidase CueO, controlled by the cytoplasmic sensor/regulator CueR, to maintain the envelope–Cu homeostasis under aerobiosis and on the CusR/CusS-controlled CusCFBA efflux system to get rid of the Cu excess from this compartment when oxygen is absent, a condition in which the oxidase is not active (Outten et al., 2001; Quintana et al., 2017; Checa et al., 2021). We showed that *Salmonella* CueP fulfils similar roles than the *E. coli* CusCFBA system in alleviating Cu stress under anaerobic conditions in their innate bacterial hosts (Pontel and Soncini, 2009; Pontel et al., 2010; Pezza et al., 2016). When overexpressed in *E. coli*, CueP partially complements a Δ*cus* mutant (Pontel and Soncini, 2009), although this periplasmic Cu chaperone with a putative Cu^2+^ reductase activity seems not to be a structural homolog of the CusCFBA system. The transcriptional activation of these Cu resistance determinants also differs but has some common features. The expression of CusCFBA occurs after the detection of surplus Cu by the metal-specific periplasmic sensor, CusS, which, in turn, phosphorylates its coupled cytoplasmic regulator CusR, both encoded within the *cus* locus (Affandi and McEvoy, 2019). *cueP* transcription depends on the simultaneous activation of the cytoplasmic Cu sensor CueR and the envelope stress sensory system CpxR/CpxA that perceives the stress caused by Cu at the bacterial cell envelope (Pezza et al., 2016), mimicking the *E. coli* CusR/CusS-controlled *cusCFBA* induction. We recently showed that *Salmonella* lost the *cus* locus and, at the same time or probably before of that, it gained *cueP* (Checa et al., 2021). However, the reasons that lead to this genetic rearrangement remains unknown.

In this work, we re-introduced the *E. coli cus* locus into the genomic place where ancestral *Salmonella cus* remnants were detected and demonstrated that the *cusCFBA* operon is expressed in response to Cu and provides resistance to this metal to the recombinant strain (Figures 1–4). The expression of this operon requires the presence of the CusR/CusS two-component system because no Cu-driven transcriptional induction was detected using the pP*cusC*-*gfp* plasmid carrying the *gfp* reporter gene under the control of the *cusC* promoter (Supplementary Figure 2). Both the indigenous *cueP* gene and the transgenic CusR/CusS-controlled *cusCFBA* operon were transcriptionally induced in response to Cu (Figures 2, 3). However, transcription from the *cueP* promoter remained active over time, even when the bacteria were well into the stationary phase, while the P*cusC* promoter was only transiently induced during the exponential phase (Figure 3). In fact, a reduction in expression of both the GFP reporter from the P*cusC-gfp* promoter or CusA-3xFLAG from the chromosomal *cusA*-3xFLAG fusion gene was evident when the bacteria reached the stationary phase. Furthermore, its Cu activation is reduced as the concentration of the metal ion increases in the culture medium. These differences could be attributed by the outcome of the metal ion, although much work is necessary to understand the role of CueP in *Salmonella*. Importantly, the simultaneous presence of both systems influenced the expression of each other (Figure 3B), demonstrating that both contribute to alleviate the toxicity caused by the metal ion in the cell envelope when bacteria grow under standard laboratory conditions.

The *cus*+ *Salmonella* transgenic strain shows wild-type resistance to Cu under aerobic conditions and an increased resistance to Cu under anaerobic conditions (Supplementary Table 3), where the multicopper oxidase CueO is inactive (Espariz et al., 2007; Pontel et al., 2010). A similar phenotype was observed in Δ*cueO* cells grown aerobically (Supplementary Figure 5) that are highly sensitive to Cu, with an exacerbated envelope stress under these conditions (Pontel et al., 2010). This is in agreement with recent *Salmonella* isolates from Cu rich environments that harbor accessory Cu resistance determinants such as Cus-like efflux pumps as well as P-type ATPases and/or periplasmic copper binding proteins encoded in plasmids as well as in other mobile genetic platforms (Mourão et al., 2016; Mastrorilli et al., 2018; Murase et al., 2018; Zhao et al., 2018; Arai et al., 2019; Branchu et al., 2019). Among them, an extrachromosomally encoded *cus* locus was present in clinic isolates (Wiesner et al., 2016). The plasmid harboring this locus also contains genes for tolerance/resistance to mercury, arsenic, and other metals and antimicrobials, indicating a link between metal and antibiotic resistance as well. The importance of these accessory Cu resistance determinants to ameliorate *Salmonella* fitness in animals that are exposed to large amounts of copper as feed supplement is clear (Mourão et al., 2015, 2016; Branchu et al., 2019). However, their relevance for virulence and, in particular, for intracellular replication of the pathogen is elusive and a matter of current investigation in different laboratories.

In contrast to the conserved arrangement of inner membrane P-type transporters and cytoplasmic Cu chaperones present in all proteobacteria to cope with Cu toxicity in the cytoplasm, different species/strains evolved specific traits to control envelope–Cu homeostasis (Giachino and Waldron, 2020). Particularly for *Salmonella*, this compartment is the main receptor for all the recent horizontally acquired genetic elements encoding Cu resistance factors (Checa et al., 2021). In this work, we showed that, at least for macrophage survival, *cueP* acquisition into the *Salmonella* genome cannot be substituted by the ancestral *cus* locus (Figure 5). Although the contribution of the efflux pump to Cu resistance in abiotic environments is clear, particularly under anaerobic conditions (Figure 4), it does not favor fitness in the Cu-rich, oxidative intracellular niche (Figure 5). Based on these, it can be speculated that the loss of *cus* from this pathogen occurred because there was no selection pressure to keep it, as *Salmonella* would rarely encounter high levels of Cu in an anoxygenic environment. Alternatively, the presence of an efflux pump in the confined space of the SCV is disfavored because the expelled toxic Cu ions rapidly re-enter the bacterial cell, resulting in a futile cycle. Therefore, in this intracellular niche, CueP Cu^2+^ binding (Osman et al., 2010) or its proposed Cu^2+^ reductase activity (Yoon et al., 2014) limits the availability of free Cu ions to exacerbate redox stress in the periplasm (Checa et al., 2021). In this context, the role of CueP as a Cu chaperone providing the metal ion to other ROS-detoxifying enzymes like SodCI and SodCII (Ladomersky et al., 2017) would be also important. In either case, it is clear that the *cueP* gene product was preserved during the evolution of *Salmonella*. Therefore, it emerges as a putative target for anti-virulence therapies to control animal and human salmonellosis.

## Data Availability Statement

The original contributions presented in the study are included in the article/Supplementary Material, further inquiries can be directed to the corresponding authors.

## Author Contributions

SC and FS contributed to the conception and design of the study. AM, JM, ME, and IT performed the experiments and analyzed the results. AM, JM, SC, and FS wrote the first draft of the manuscript, contributed to manuscript revision, and read the submitted version. All authors contributed to the article and approved the submitted version.

## Conflict of Interest

The authors declare that the research was conducted in the absence of any commercial or financial relationships that could be construed as a potential conflict of interest.

## Publisher’s Note

All claims expressed in this article are solely those of the authors and do not necessarily represent those of their affiliated organizations, or those of the publisher, the editors and the reviewers. Any product that may be evaluated in this article, or claim that may be made by its manufacturer, is not guaranteed or endorsed by the publisher.
