# Supplementary material for: Evolution of Copper Homeostasis and Virulence in Salmonella
Source: Front Microbiol. 2022 Mar 16;13:823176. doi: 10.3389/fmicb.2022.823176 (PMC8966772; doi:10.3389/fmicb.2022.823176)
Supplement: Supplementary file 1 [file Data_Sheet_1.PDF]

Supplementary Material

Evolution of copper homeostasis and virulence in *Salmonella*

Andrea A. E. Méndez, Julián I. Mendoza, María Laura Echarren, Ignacio Terán, Susana K. Checa*, Fernando C. Soncini*

Instituto de Biología Molecular y Celular de Rosario, Facultad de Ciencias Bioquímicas y Farmacéuticas, Universidad Nacional de Rosario, Consejo Nacional de Investigaciones Científicas y Técnicas, Rosario, Argentina

Supplementary Table 1. Bacterial strains and plasmids used in this study.

Strain	Relevant genotype or properties	Refs. or source
Eschericha coli (EC)		
W3110	F- λ- rph-1 INV(rrnD, rrnE)	(Bachmann, 1972)
PB11797	W3110 *cusS*-Cm^R *cusA*::3xFLAG-Km^R	Laboratory stock
Salmonella enterica serov. Typhimurium (STM)		
14028s	Wild type	ATCC®-14028™
PB13957	*cusRS*-Cm^R *cusCF* #2	This work
PB14006	*cusRS*-Cm^R *cusCFBA*::3xFLAG-Km^R #8	This work
PB6127	Δ*cueP*-Cm^R	(Pontel and Soncini, 2009)
PB6128	Δ*cueP*	(Pontel and Soncini, 2009)
PB10942	*cueO*::3xFLAG-Km^R	Laboratory stock
PB14907	Δ*cueP cusCFBA*::3xFLAG-Km^R	This work
PB9970	*cueP*:3xFLAG	(Pontel and Soncini, 2009)
PB14960	*cueP*:3xFLAG *cusCFBA*::3xFLAG-Km^R	This work
PB11747	Δ*phoP*	Laboratory stock
PB7937	Δ*cueO*	Laboratory stock
PB14763	Δ*cueO cusCFBA*::3xFLAG-Km^R	This work
PB8032	Δ*cueO* Δ*cueP*	Laboratory stock
PB14764	Δ*cueO* Δ*cueP* *cusCFBA*::3xFLAG-Km^R	This work
PB9326	ΔgolT ΔcopA	Laboratory stock
PB14765	ΔgolT ΔcopA *cusCFBA*::3xFLAG-Km^R	This work

Plasmid	Relevant genotype or properties	Refs. or source
pKD46	$oriR_{pSC101}$ts P_{araB} *exo-bet-gam* Amp^R	(Datsenko and Wanner, 2000)

pPROBE-OT	*repp* BBR1 Sp^R promoter-less *gfp*	(Miller et al., 2000)
pP*cueP-gfp*	pPROBE-OT derived plasmid carrying the *gfp* gene under the control of the CueR-dependent *cueP* promoter	This work
pP*cusCFBA*-*gfp*	pPROBE-OT derived plasmid carrying the *gfp* gene under the control of the CusRS-dependent *cusCFBA* promoter	This work
p*cueP*	pUH21-2 $lacI^q$ derived plasmid carrying *cueP*	(Pontel and Soncini, 2009)

Supplementary Table 2. Oligonucleotides

Primer name	Sequence (5´-3´)	Purpose
Cus SF P1 Fw	TTTGCTGGCTAGTGATAGCGCC TTTCACTATCGCTACAGTCGCT GGTTATGTTTAGGGTG	Amplification of the *Cm-cusRS cusCF* fragment for λ Red insertion
Cus SF P2 Rv	AACCCGCCTGCAGTTTGACGCC GCAGGCGGGGTGCACACGAAC CTGGGTTACTGGCTGAC	Amplification of the *Cm-cusRS cusCF* fragment for λ Red insertion
P1 Fwd CusA FLAG Km	AAACCGGCGACAAAGTGGCG	Amplification of the cus*BA*::3xFLAG-*km* fragment for λ Red insertion
Cus FA P2 Rv	AACCCGCCTGCAGTTTGACGCC GCAGGCGGGGTGCACACGCGC AAGACACAATCCACACG	Amplification of the cus*BA*::3xFLAG-*km* fragment for λ Red insertion
RevP1	CGAAGCAGCTCCAGCCTACAC	PCR checking of the *Cm-cusRS cusCF* fragment
cusA Fwd	TCAGCGAGCAGAAGCTGG	PCR checking of the *Cm-cusRS cusCF* fragment
P2 Fwd	TAAGGAGGATATTCATATG	PCR checking of the cus*BA*::3xFLAG-*km* fragment
Cus-pKNG101 P2 Rv- BamHI	CGCGGATCCATAATCGCCACG CGCC	PCR checking of the cus*BA*::3xFLAG-*km* fragment
promCueP-Long-Fw BglII	AGAGATCTACGTAACCAGCCT GTAAG	Cloning of the *cueP* promotor gene in pPROBE-OT´– SmaI restriction site
promcueP-Rv	AGCCCGGGCAAAAGTAATAAC CATGATG	Cloning of the *cueP* promotor gene in pPROBE-OT´– SmaI restriction site
Fw-HindIII-cusR	CCCAAGCTTAACCGGCTTCGGT TAACCC	Cloning of the *cusA* promotor gene in pPROBE-OT´– HindIII restriction site
Rv-EcoRI-cusC	CCGGAATTCGGGCCACACAAA ATGGCAG	Cloning of the *cusA* promotor gene in pPROBE-OT´– EcoRI restriction site

Supplementary Table 3. Cu-resistance

Strain	MIC (mM)	
	**+O_2**	**-O_2**
wt	4	0.3
cus+	4	1
Δ*cueP*	4	0.15
Δ*cueP cus+*	4	1
Δ*goltT* Δ*copA*	1	ND
Δ*golT* Δ*copA cus+*	1	ND
Δ*cueO*	2	ND
Δ*cueO cus+*	4	ND
Δ*cueO* Δ*cueP*	1	ND
Δ*cueO* Δ*cueP cus+*	4	ND

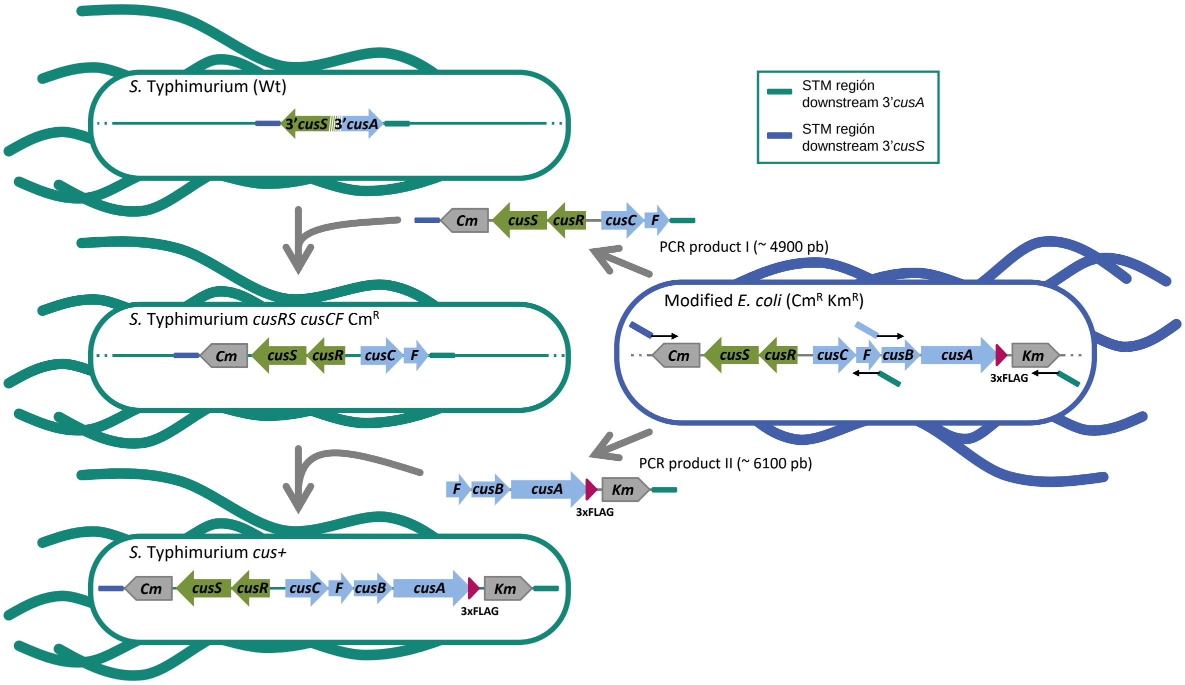

Supplementary Figure 1. Schematic representation of the construction of the *cus*+ *S.* Typhimurium 14028s transgenic strain, harboring the *E. coli* W3110 *cus* locus inserted into the *S.* Typhimurium *cus* scar. PCR amplification of a ~4900 pb fragment containing the Cm^R cassette as well as the *cusRS* operon, *cusC* and *cusF*, and a ~6100 pb fragment containing the Km^R cassette as well as *cusF*, *cusB* and *cusA*::3xFLAG, were performed using a recombinant *E. coli* W3110 harboring a Cm^R and a Km^R cassettes inserted downstream of *cusS* and *cusA*, respectively. These fragments were sequentially inserted into *S.* Typhimurium 14028s chromosome by the one-step procedure (Datsenko and Wanner, 2000) to render the *cus*+ transgenic strain.

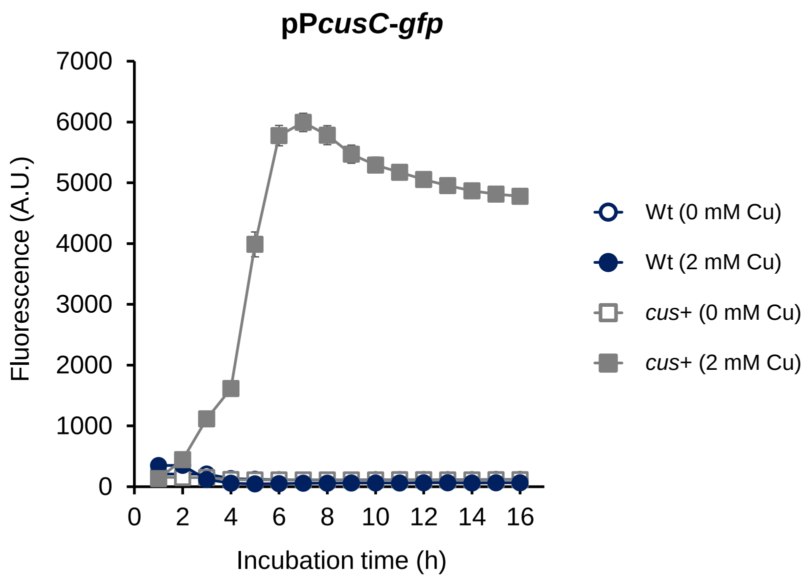

Supplementary Figure 2. Transcription from the P*cusC* promoter requires the CusR/CusS two-component system. Kinetic analysis of the Cu-induced fluorescence from the wild-type and the *cus*+ strains harboring *gfp* transcriptional fusion to the P*cusC* promoter, as indicated. Cells were grown either in LB or LB supplemented with 2 mM $CuSO_4$ (as indicated). Emitted fluorescence and $OD_{600\ nm}$ were recorded every hour for 16 h. Normalized fluorescence was expressed as arbitrary units (A.U). The data correspond to mean values of three independent experiments performed in duplicate. Error bars represent SD.

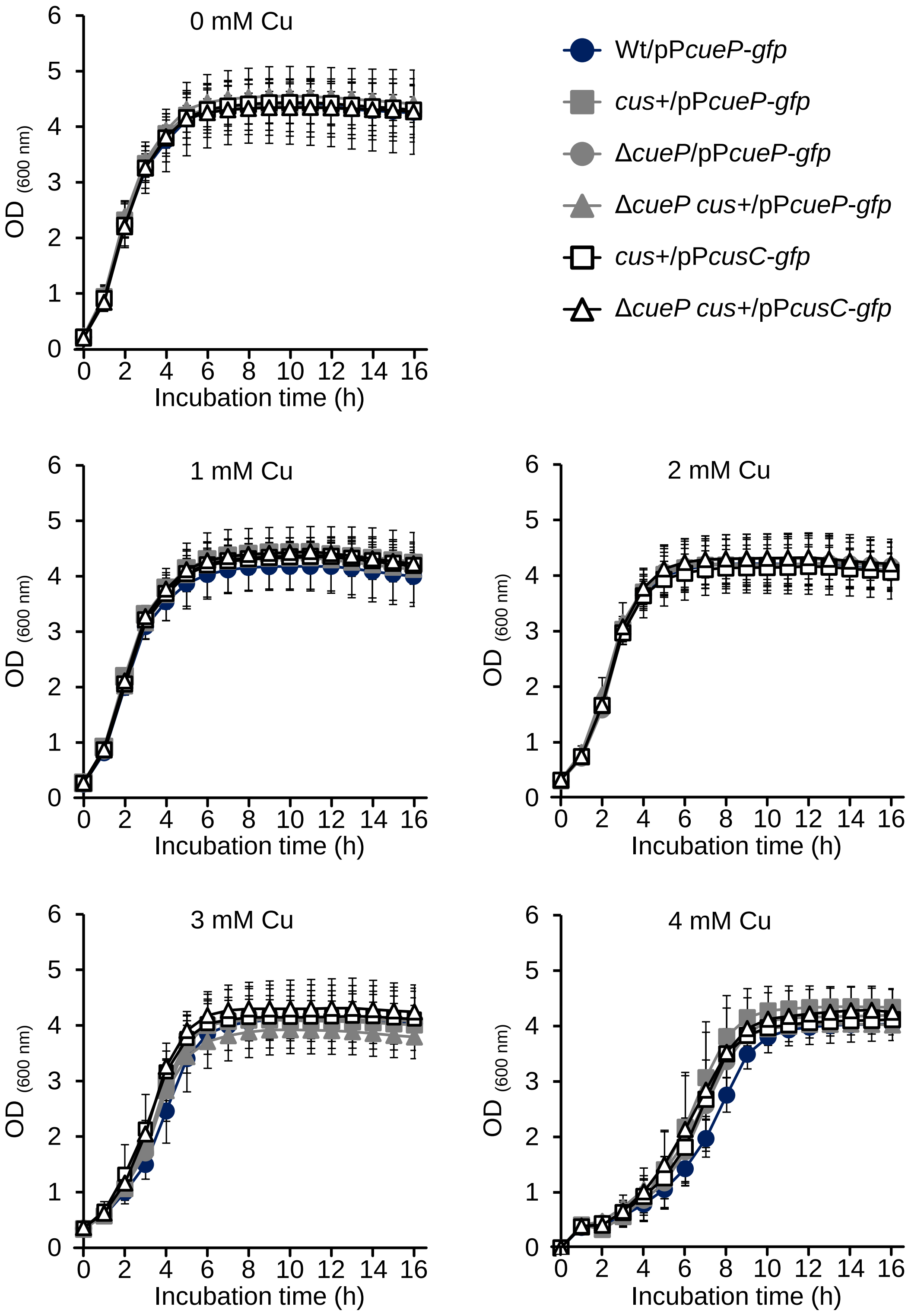

Supplementary Figure 3. $OD_{600\ nm}$ of the strains tested in the experiment shown in Fig. 3. The data correspond to mean values of at least three independent experiments performed in duplicate. Error bars represent SD.

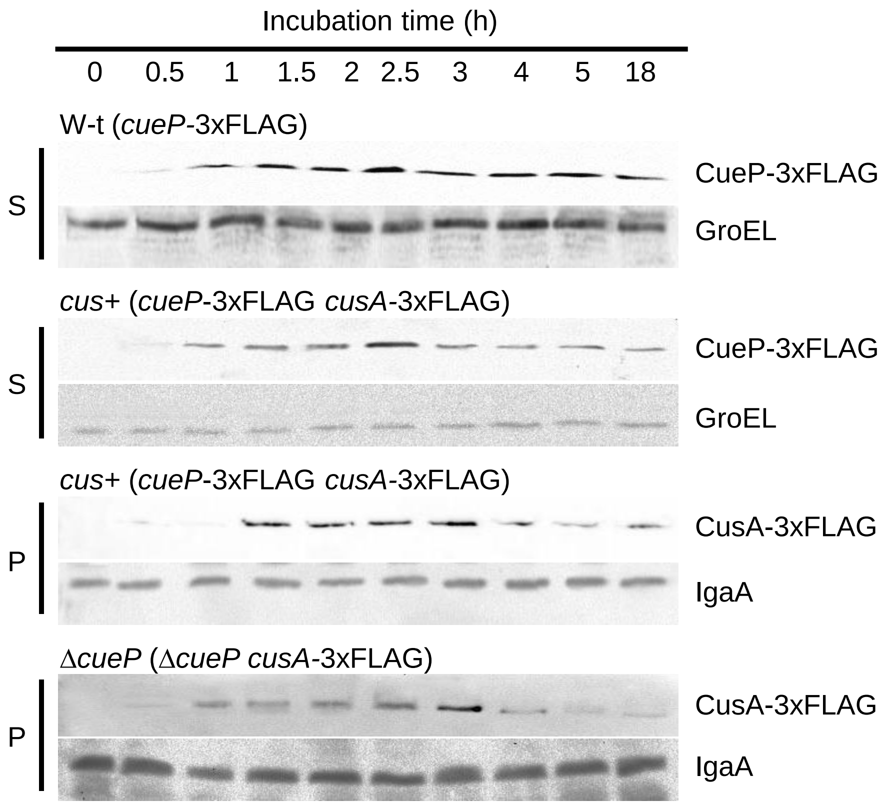

Supplementary Figure 4. Representative western blot analysis for the detection of CueP-3xFLAG or CusA-3xFLAG from the *cueP*-3xFLAG (W-t), the *cus*+ *cueP*-3xFLAG *cusA*-3xFLAG (*cus*+), or the *cus*+ *cusA*-3xFLAG Δ*cueP* (Δ*cueP*) strains after the addition of Cu-induced expression. 20 µg of total protein cell extracts from cells grown in LB with the addition of 2 mM $CuSO_4$ was analyzed by SDS/PAGE, followed by transfer to nitrocellulose and development using monoclonal anti-FLAG antibodies. CueP or CusA relative levels were normalized to GroEL or to IgaA, using rabbit IgG anti-GroEL, or anti IgaA.

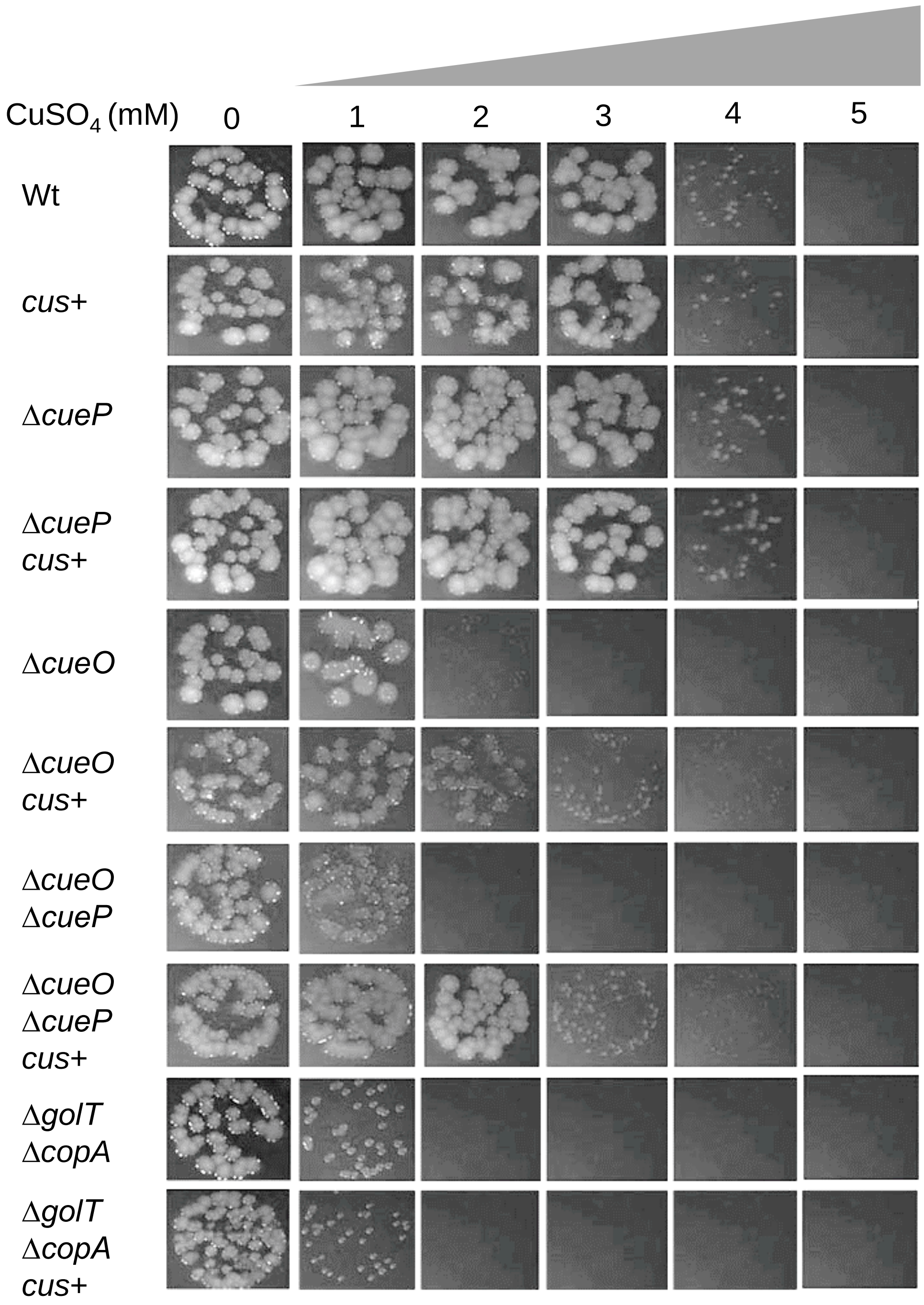

Supplementary Figure 5. *cus* is dispensable for *Salmonella* Cu-resistance under aerobic conditions but contributes to alleviate toxicity in cells lacking *cueO*. Comparative copper-sensitivity assays of the wild-type (Wt) or the indicated mutant or transgenic strains in LB-agar plates supplemented with $CuSO_4$. (-) indicate no metal addition. 10 µl-aliquots from a 10^{-6} dilution in PBS of overnight cultures were applied on top of LB agar plates supplemented with the indicated $CuSO_4$ concentrations. After incubation at 37°C for 24 h the plates were photographed. The data correspond to representative images of at least three independent experiments done in duplicate.

References

Bachmann, B.J. (1972). Pedigrees of some mutant strains of Escherichia coli K-12. Bacteriological reviews 36, 525-557.

Datsenko, K.A., and Wanner, B.L. (2000). One-step inactivation of chromosomal genes in Escherichia coli K-12 using PCR products. Proc Natl Acad Sci U S A 97, 6640-6645.

Miller, W.G., Leveau, J.H.J., and Lindow, S.E. (2000). Improved gfp and inaZ Broad-Host-Range Promoter-Probe Vectors. Molecular Plant-Microbe Interactions® 13, 1243-1250.

Pontel, L.B., and Soncini, F.C. (2009). Alternative periplasmic copper-resistance mechanisms in Gram negative bacteria. Mol Microbiol 73, 212-225.
